# The Impact of Child Emotional and Behavioral Difficulties on Caregivers’ Mental Health: Insights from Families of Children Orphaned by HIV in Southern Uganda

**DOI:** 10.1007/s40609-026-00453-6

**Published:** 2026-03-12

**Authors:** Melody Rachel Konadu Frempong, Vicent Ssentumbwe, Josephine Nabayinda, Flavia Namuwonge, Samuel Kizito, Proscovia Nabunya, Fred Ssewamala

**Affiliations:** 1 Washington University in St. Louis, St Louis, USA

## Abstract

**Background:**

In Uganda, children orphaned by HIV are at a high risk of emotional and behavioral difficulties (EBDs). This elevated risk of EBDs has implication on caregivers’ mental health given that caregiving is an involving responsibility that can affect mental, physical health and overall well-being. While the existence of child EBDs and its impact on caregivers’ mental health is well documented in high-income countries, few studies in Sub-Saharan Africa have examined the impact of child emotional and behavioral difficulties on caregiver mental health among families of children orphaned by HIV. This study aims to examine the relationship between child difficulties and caregiver mental health in Southern Uganda.

**Methods:**

Utilizing baseline data from the Suubi-Maka study (*N* = 346 dyads), a two-arm cluster-randomized controlled trial, the current study examines the relationship between children’s EBDs and caregiver mental health. Child EBDs were measured using the Child Strengths and Difficulties scale as reported by caregivers. Caregiver mental health was measured by the Brief Symptom Inventory. Mixed effect linear regression model was used to examine the relationship between child difficulties and caregiver mental health. Control variables were caregiver age, gender, household size, family size, social support, family cohesion, family assets, and substance use (alcohol and cigarettes).

**Results:**

The mean age of the caregivers was 45.78 years. Results showed a significant positive association between child EBDs and caregiver mental health problems. High levels of child difficulties were associated with poorer caregiver mental health functioning (β = *0.85*, 95% CI = *0.64, 1.06, p* < *0.001*). Additionally, being a male caregiver (β = 10.18, 95% CI = *5.65, 14.72, p* < *0.001*), and alcohol use (β = *5.22*, 95% CI = 2.03, 8.41, *p* = 0.001) were associated with poorer caregiver mental health. Notably, there was also a significant negative association between family cohesion and caregivers’ mental health problems (β=−*0.57*., 95% CI = −*1.05*, −*0.09*, *p* = −0.019).

**Conclusion and Implication:**

The results emphasize the complex interplay of child difficulties, family cohesion, gender, and their influence on caregivers’ mental health, posing a significant implication for global health. The findings suggest caregiving may not be exclusively undertaken by women, highlighting the importance of considering the mental health needs of male caregivers in the development of policies and support services. Future research is needed to explore the longitudinal pathways of this relationship: whether improving child EBDs can improve caregiver mental health or vice versa. The result further indicates the need to build innovative and multifaceted interventions to address caregiver mental health challenges in SSA.

## Introduction

Sub-Saharan Africa (SSA) remains disproportionately affected by HIV, with over 25 million people living with the virus and hundreds of thousands of new infections and HIV-related deaths reported annually (WHO, 2024).). In 2022, almost 760, 000 newly infected cases were recorded and 380,000 were reported to have died from HIV/AIDS related illness. Uganda is one of the countries which is worst affected by the HIV epidemic, with an estimated number of 1.5 million adults (15 and above) living with HIV (UNAIDS, 2024). Although the prevalence rates of HIV/AIDS have been declining in recent years due to the availability of anti-retroviral therapy (ART), the number of children (between 0 and 17 years) who have lost one or both parents to HIV is still high with about 870, 000 in 2023 (UNAIDS, 2024).

Children orphaned by HIV are at a high risk of emotional and behavioral difficulties (EBDs) (Asanbe et al., 2016). Irrespective of the cause of the loss of a parent, the loss can have a drastic effect on a child. Importantly, the grieving process coupled with the shame and stigma surrounding HIV can heighten the emotional problems of a child (Rukunga, 2022). These emotional burdens can be demonstrated through hyperactivity, anxiety and conduct problems. Studies have consistently shown that children orphaned by HIV as well as those made vulnerable by HIV/AIDs are at high risk of (Cluver et al., 2013)anxiety, PTSD, depression, relationship difficulties and anger issues (Gamarel et al., 2017; Hermenau et al., 2015; Narh Doku et al., 2019; Sharp et al., 2015).

A lot of factors account for these emotional problems among HIV orphaned children. For instance, children orphaned by HIV are likely to face financial challenges(Cluver et al., 2013; Tutlam et al., 2023) hence reducing their access to education, basic needs and healthcare increases their vulnerability to emotional and behavioral problems. In other instances, some children often assume the role as caregivers to their parent during their sickness (Piot et al., 2007) creating an experience of trauma from witnessing the decline and the death of their parents. While, others also stand the risk of experiencing abuse, neglect and exploitation (Doku et al., 2015; Hermenau et al., 2015; Narh Doku et al., 2019), which all increases their vulnerability to emotional and behavioral difficulties.

Caregivers who are mostly grandparents, foster guardians, or extended relatives have to navigate the dual pressure of meeting children’s complex needs while navigating their own grief and social stigma surrounding HIV. In low resource setting, Caregiving is fundamental to the well-being of children, especially within a context where children are more vulnerable to diseases such as HIV/AIDs. In sub-Saharan Africa (SSA), almost 10.3 million children are orphaned by AIDS and remain in need of support (Cluver et al., 2023). Caregivers are mostly family members who assume the role of parents, provide care and the needs of children orphaned by HIV. The huge number of children who are orphaned due to HIV places an enormous burden on the extended families (Mpofu & Tshabalala, 2021); Karimli et al., 2012; Kidman & Thurman, 2014). Navigating through the caregiving responsibilities goes beyond providing basic needs such as shelter, food and medical care as it entails providing emotional support and navigating through emotional and other complexities, especially among children who might have experienced illness, trauma abuse or neglect (Elias et al., 2018). As a result, caregivers may experience psychological distress and caregiver burnout. Caregivers may experience psychological distress and caregiver burnout, which can also compromise the quality of care provided to children.

The burden of caregiving is well documented. For instance, evidence suggests that many caregivers experience anxiety, stress and depression (Casale et al., 2015; Spielman et al., 2021). Additionally, caregivers of orphaned children may experience financial difficulties coupled with ongoing grief due to the loss of a family member (Bhana et al., 2010). Studies have documented feelings of isolation and unsupportiveness among caregivers, leaving little or no room for social engagement or self-care (Kayaalp et al., 2021; Vasileiou et al., 2017). Notably, caregivers in low-resource settings such as sub-Saharan Africa are mostly faced with additional challenges such as lack or limited access to health care, stigma and financial constraints which aggravate their mental health and stress (Adelman et al., 2014; Daliri et al., 2024; Gumikiriza-Onoria et al., 2024; Ndlovu & Mokwena, 2023). These psychosocial, economic, and structural challenges contribute to caregivers’ mental health difficulties, with heightened vulnerability among those caring for children orphaned by HIV.

HIV-related parent death has a significant and long-term impact on a child’s behavioral, psychosocial, and emotional outcomes (Li et al., 2015), and this requires a high level of support. Providing care to children with EBDs pose an additional source of stress and strain for parents and caregivers(Green et al., 2020). Evidence suggest that EBDs among children are associated with stress in parenting ((Green et al., 2020);(Carroll et al., 2024). The distinct physiological, emotional, and physical needs of children with EBDs place additional stress on caregivers, yet this remains underexplored. Existing studies on the mental health of caregivers of children orphaned by HIV in sub-Saharan Africa (Jardin et al., 2017; Kuo et al., 2012; Kuo & Operario, 2011) have largely focused on reporting depression and alcohol use, overlooking other caregiving challenges, including managing children’s emotional and behavioral difficulties. Despite evidence on caregiver mental health among HIV-affected families, limited research has explored the mental health of caregivers providing care for children exhibiting emotional and behavioral difficulties. For a long time, the literature on caregiver mental health in SSA settings has focused on caring for children with developmental disabilities (Chukwuemeka & Obioha, 2024), cancer (Bekui et al., 2023; Namazzi et al., 2019) and neurological disorders (Ademosu et al., 2021). Studies focused on caring for children orphaned by HIV are limited. This particularly warrants attention as caregiver wellbeing directly shapes the quality of care provided to orphaned children and influences their developmental and mental health outcomes.

Therefore, the present study seeks to examine the relationship between EBDs among children orphaned by HIV and the mental health of their caregivers. By examining the relationship between child EBDs and caregiver mental health within HIV-affected families, this study contributes new empirical evidence to a largely underexplored area. The findings advance theory by demonstrating that attachment disruptions and family system dynamics jointly shape caregiver well-being in high-stress and low-resource contexts. The study also offers practical guidance for designing interventions that support both caregivers and children. This study provides a foundation for developing context-appropriate, family-centered strategies that can strengthen caregiver resilience and improve outcomes for children orphaned by HIV.

### Theoretical Framework

This study is guided by both Attachment Theory (Bowlby, 1969) and Family Systems Theory (Bowen, 1966) (FST), which together provide a complementary framework for understanding the relational and contextual factors shaping emotional and behavioral difficulties among children orphaned by HIV/AIDS and the mental health of their caregivers.

Attachment theory posits that early relationships with caregivers play a central role in shaping a child’s emotional regulation, behavioral functioning, and psychological development across the life course (Bowlby, 1969). Disruptions in attachment, such as the loss of a biological parent, may undermine a child’s sense of security and increase vulnerability to emotional and behavioral difficulties. When children exhibit such difficulties, caregivers may experience heightened stress, emotional burdens, and feelings of inadequacy, contributing to psychological strain. In turn, caregivers’ mental health challenges may compromise their capacity to provide consistent and sensitive care, reinforcing a reciprocal cycle of distress between child and caregiver functioning.

Family Systems Theory extends this relational perspective by conceptualizing the family as an interconnected emotional system in which the functioning of each member influences, and is influenced by, others (Bowen, 1966; Hampton-Anderson et al. 2025). Within this framework, child emotional and behavioral difficulties are understood as individual outcomes that are embedded within broader family dynamics, including caregiving roles, communication patterns, emotional support, and family cohesion (Ssewamala et al., 2023). Family stressors such as parental loss due to HIV/AIDS, financial strain, and role re-organization can disrupt family stability and amplify psychological vulnerability among children and their caregivers.

**Fig. 1:**
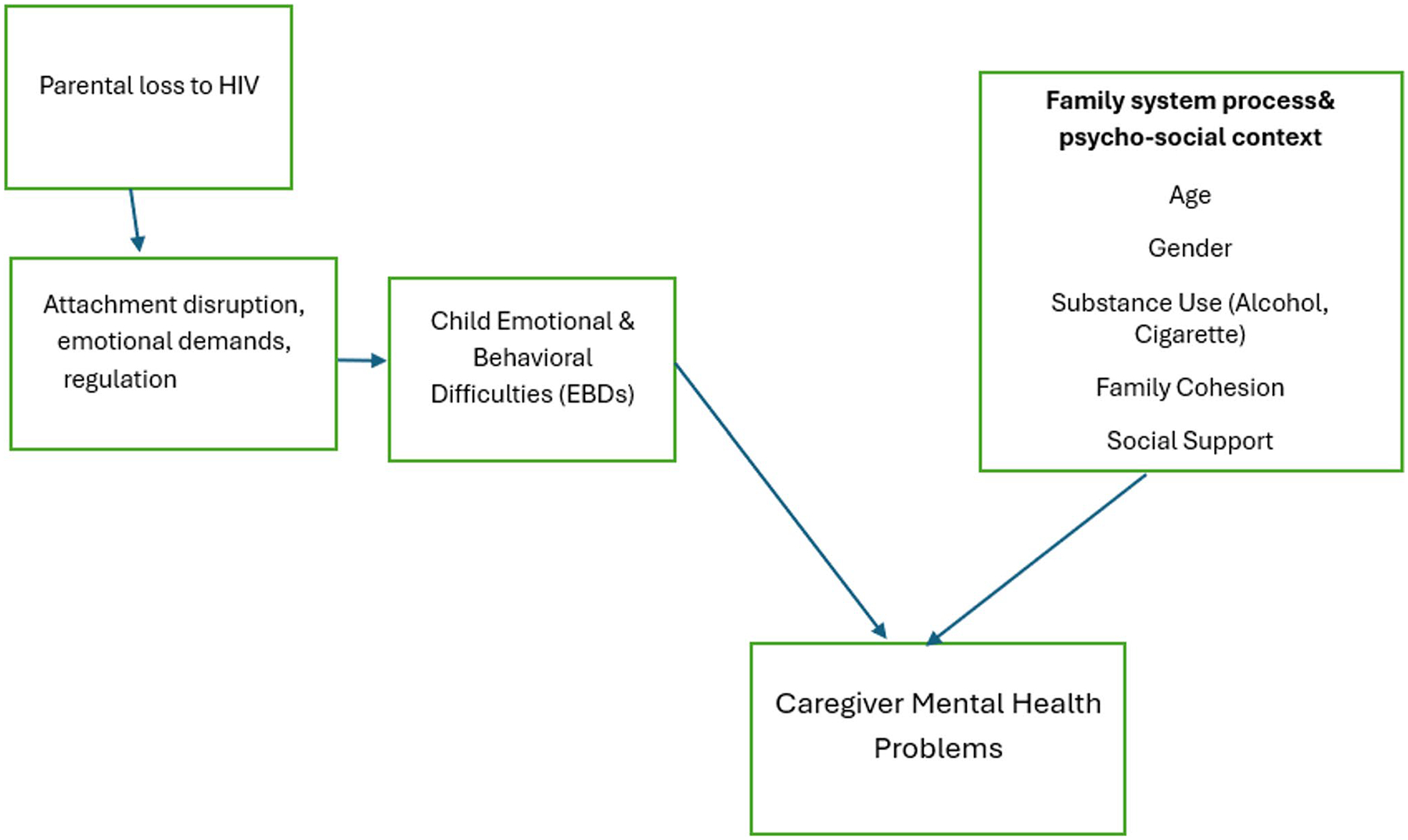
Conceptual framework

Importantly, these attachment and family processes are situated within collectivist African family systems, where caregiving responsibilities are frequently shared among extended family members, including grandparents, aunts, uncles, and older siblings. In the Ugandan context, parental loss due to HIV/AIDS often necessitates shifts in caregiving arrangements, which may affect the consistency and quality of emotional bonds as well as overall family functioning. From an integrated attachment and family systems perspective, caregiver mental health is understood both as an outcome of caregiving stress and as a product of ongoing relational and systemic strain within the family unit. In the context of this study, the loss of a parent due to HIV disrupts the child’s attachment system, leading to grief, anxiety, and emotional and behavioral issues. Caregivers who step in may struggle to provide secure attachment due to parenting stress, their own mental health challenges, financial stress, or lack of resources. Guided by these theories, the present study conceptualizes child emotional and behavioral difficulties as relational stressors that both reflect and shape caregiving dynamics within the family system, with important implications for caregivers’ mental health outcomes. Figure 1 illustrates the conceptual framework of this study, grounded in attachment and family systems theory.

## Methods

### Study Design

We analyzed baseline data from the Suubi-Maka study (2008–2012), a two-arm cluster-randomized study. Detailed information about the design and implementation of the intervention has been published separately (Ssewamala et al., 2008). The Suubi Maka is a Luganda phrases translated as *hope for families*. The aim of the Suubi-Maka study was to explore an economic empowerment intervention among poor families who are the primary care providers to children orphaned by HIV/AIDS.

### Sampling and Participants

The study recruited 346 child-caregiver dyads from 10 rural public Schools in the Masaka and Rakai districts of South-western Uganda (Osuji et al., 2018; Tutlam et al., 2023). Randomization was done at the school level. The two districts are known to be heavily affected by HIV and AIDS. Children were eligible for enrollment in the study if they were (1) aged 10–17 years; (2) had lost at least one biological parent to HIV/AIDS; (3) registered in the last two years of primary school (6th and 7th grade in US schools) and (4) living in a household. Caregivers were eligible for the study if they were aged 18 or older and reported caring for the enrolled child. For this paper, the sample used was derived from the parent/caregiver questionnaire.

### Ethical Consideration

Participating in this study was voluntary. Caregiver-informed consent and child assent were obtained from caregivers and children, respectively, before they participated in the study. The consent and assent forms were translated into Luganda, and caregivers and children completed the forms separately to avoid coercion. Study procedures were approved by Columbia University Institutional Review Board (AAAD2525) and the Uganda National Council for Science and Technology (ref SS 1540). Several measures were implemented to ensure privacy, confidentiality, and participant safety during data collection. Informed consent and assent procedures, as well as all interviews, were conducted separately for children and caregivers to minimize coercion, reduce power dynamics, and promote candid responses. Each interview took place in a private, secluded setting where only the participant and the trained interviewer were present. Unique study identification numbers were used in place of personal identifiers on all data collection tools, and completed questionnaires were securely stored with access limited to authorized study personnel. With respect to participant well-being, all interviewers received training in ethical research conduct and in recognizing signs of severe emotional, behavioral, or mental health distress.

## Measures

### Outcome Variable

The outcome variable for this study is primary caregiver mental health, which was assessed using the Brief Symptom Inventory (Derogatis, 1993). This scale has been used in other studies in similar settings and has demonstrated good psychometric properties (Kagotho & Ssewamala, 2012; Ssewamala et al., 2008). The 34-item scale assesses nine symptom dimensions, including somatization, obsessive–compulsive, interpersonal sensitivity, depression, anxiety, hostility, phobic anxiety, paranoid ideation, to psychoticism (e.g., “Feeling no interest in things”, “Feeling hopeless about the future”, “Feeling easily annoyed or irritated”, “Feeling that you are watched or talked about by others”, “Poor appetite”, “Pains in heart or chest”,.). Each item was rated on a 5-point Likert scale from never (1) to always (5). A summated score was calculated across the 34 items, with higher scores indicating greater levels of mental health distress (Cronbach’s alpha = 0.89).

### Independent Variables

The main independent variable, EBDs was measured using Child Strength and Difficulties (SDQ) (Goodman, 1997), as reported by caregivers. The (SDQ) is a 25-item measure comprising five subscales: conduct problems, peer problems, emotional problems, hyperactivity, and pro-social behavior of their children. The Likert responses ranged from 1 to 5 with 1 = Never, 2-rarely, 3 = sometimes, 4 = most of the time and 5 = always. Sample items include: *Your child is restless*, *overactive*, *cannot stay still for long*, *your child would rather be alone than with other teenagers*, *Your child often fights with other youth or bullies them*. A summated score of the 25 items were created and 10 items in opposite direction were reverse coded for higher scores to represent higher levels of child difficulties (Cronbach’s alpha = 0.64). Internal consistency was modest but acceptable, consistent with prior validation of the SDQ in similar Ugandan contexts (Thumann et al., 2016).

### Control Variables

Several variables were included as controls in the analysis. These included caregiver age and gender, household size, and household asset ownership. Asset ownership was assessed using a 17-item index in which caregivers were asked whether they owned specific assets such as a house, rental property, car, coffee garden, or banana garden. Each asset was coded as 1 if owned and 0 if not owned, and responses were summed to generate a total household asset score. Family cohesion was measured using a six-item scale assessing the degree of commitment, help, and support among family members (α = 0.71) (Moos & Moos, 2002; Skinner et al., 2009), with items rated on a five-point Likert scale ranging from 1 (“never”) to 5 (“always”). Social support networks were assessed by asking caregivers to identify two individuals who were important and supportive to the child, and to rate the level of support using ten items on a five-point Likert scale (1 = “never” to 5 = “always”) (α = 0.83); sample items included availability when the child needs support and making the child feel good about himself or herself. Caregiver cigarette use and alcohol use were also included as control variables and were coded dichotomously (1 = yes, 0 = no).

### Data Analysis

Data analysis was conducted using Stata version 18.0. We summarized participant characteristics using means and standard deviations for continuous variables and counts and percentages for categorical variables. Bivariate analyses assessing the correlation of each continuous predictor with the outcome, and t-tests for binary predictors with the outcome were conducted.

We used mixed-effect linear regression analysis to examine the association between children’s strengths and difficulties and their caregivers’ mental health (Zhou et al., 2010). Mixed-effects linear regression was used to account for the hierarchical structure of the data. This is particularly significant given the nested structures of the data since the participants were recruited from 10 schools. The model included two levels: study participants (level 1) nested within schools (level 2). We included random effects for schools and participants to account for variability between schools and participants, respectively. Intraclass correlation coefficients (ICCs) were computed to estimate the proportion of variance attributable to each level. We examined the models to determine that the residuals were normally distributed, there was homoskedasticity, and no multicollinearity (Variance Inflation Factors (VIFs) below 5) was detected. The intra-class correlation coefficients were determined using the calculated variances at each level. We reported beta coefficients along with their Huber–White cluster-adjusted confidence intervals. Statistical significance was established at a p-value of 0.05.

## Results

### Sample Characteristics

Table 1 presents the demographic and psychosocial characteristics of the sample. The mean caregiver age was 45.78 years (SD = 14.56), and the majority were female (79.4%). The average household size was 6.46 people (SD = 2.28). Approximately 6.7% of caregivers reported cigarette use, and 62.7% reported alcohol use. The mean score for child difficulties was 51.19 (SD = 9.24), and the caregiver’s mental health was 73.60 (SD = 19.36).

### Bivariate Analysis

Table 2 presents results from bivariate analyses. Caregivers’ mental health was positively correlated with child difficulties (*r* = 0.37, *p* < 0.001) and negatively correlated with family cohesion (*r* = −0.12, *p* = 0.023). In addition, caregivers’ mental health scores differed significantly by gender (t = −3.12, *p* = 0.002) and alcohol use (t = −2.10, *p* = 0.036).

### Association Between Children’s Emotional and Behavioral Difficulties and Caregivers’ Mental Health

Model diagnostics confirmed that the key assumptions of the mixed-effects regression were met: residuals were normally distributed, predictors showed no multicollinearity (VIFs < 5), and homoscedasticity was satisfied.

**Fig. 2:**
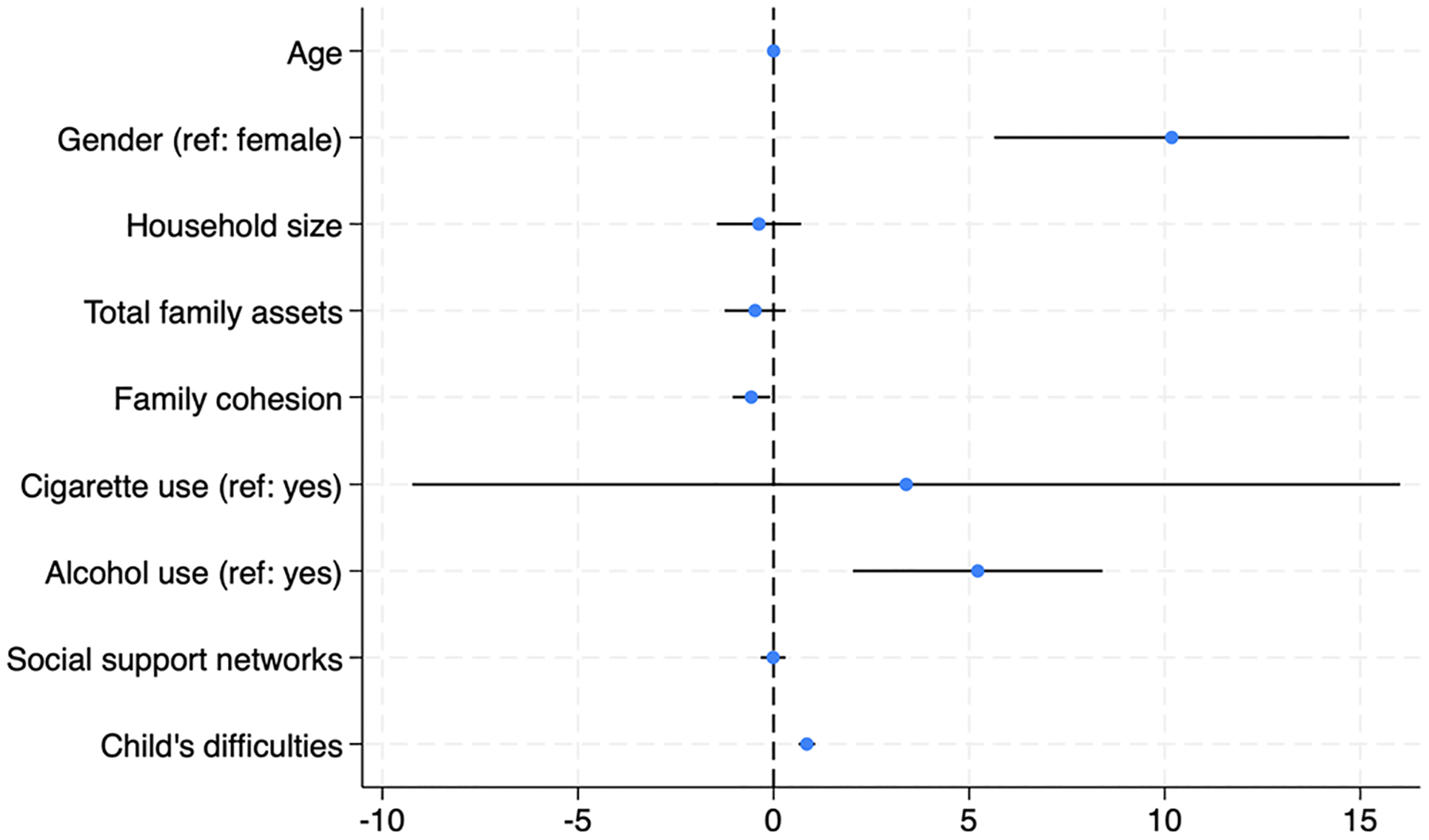
A forest plot showing the association between child’s emotional and behavioral difficulties and the caregiver’s mental health

From the mixed effect regression model in Table 3, higher levels of child difficulties were associated with higher levels of caregiver mental health problems (β = *0.85*, 95% CI = *0.64, 1.06, p* < *0.001*) after controlling for age, gender, household size, family assets, social support, family cohesion, and substance use. Other factors that were significantly associated with caregiver mental health included the caregiver’s sex, alcohol use, and family cohesion. Specifically, male caregivers had significantly poorer mental health outcomes than female caregivers (β = 10.18, 95% CI = *5.65, 14.72, p* < *0.001*). Alcohol use was associated with worse caregiver mental health (β = *5.22*, 95% CI = 2.03, 8.41, *p* = 0.001), indicating higher mental health difficulties among caregivers who reported alcohol use. In contrast, family cohesion was negatively associated with caregivers’ mental health problems (β=−*0.57*, 95% CI = −*1.05*, −*0.09, p* = −*0.019*), indicating that caregivers with stronger family cohesion are associated with improved caregiver mental health. See Table 2 for the associations between EBDs and Caregiver’s Mental Health and Fig. 1 for the graphical presentation of the model. The intraclass correlation coefficient (ICC) for schools was very low (ICC = 0.002, 95% CI: <0.001, 1.00), while the ICC for participants was high (ICC = 0.994, 95% CI = 0.968, 1.00), suggesting that nearly all variations in caregiver mental health occur between individuals rather than between schools. Figure 2 also provides a visual plot of the association between child EBDs and caregivers mental health.

## Discussion

This study examined the association between child EBDs and caregivers’ mental health among families of children orphaned by HIV. The findings indicate that caregiver mental health problems were associated with caregivers/s sex, alcohol use, and family cohesion. Specifically, male caregivers reported poorer mental health outcomes compared to female caregivers and caregivers who reported alcohol use also experienced mental health problems.

The findings support the attachment theory, which asserts that the loss of a parent disrupts a child’s attachment system, which can lead to emotional and behavioral problems (Bowlby, 1969). When caregivers themselves experience psychological distress, their emotional availability and responsiveness may be compromised, making it more difficult to maintain a secure caregiver–child bond. This is consistent with the theory, as early disruptions in caregiving relationships can destabilize a child’s attachment system, increasing vulnerability to emotional and behavioral problems. This can worsen child EBDs, reinforcing the cycle of distress within the caregiving relationship. At the same time, the results are consistent with Family Systems Theory, which emphasizes that individual distress cannot be understood in isolation but must be viewed within the broader family context. Factors such as family cohesion, gendered caregiving roles, and caregiver coping behaviors (including alcohol use) shape how stress circulates within the family system. In low-resource, HIV-affected households, these dynamics may be intensified by economic strain, stigma, and limited access to mental health services. From this perspective, interventions that strengthen family communication, reduce conflict, and enhance shared problem-solving are theoretically justified because they target the relational processes through which caregiver distress and child difficulties influence one another.

This finding also corroborates with prior research showing that caregivers of children orphaned by HIV face mental health difficulties (Mamukeyani, 2021; Nabunya et al., 2014). Notably, the substantial effect in this relationship may suggest that children with EBDs mostly require additional attention and social support. The additional attention, therefore, increases the demand for care, which can over-whelm caregivers, leading to burnout and mental distress.

Importantly, our findings also suggest a bidirectional relationship between child EBDs and caregiver mental health. Caregivers experiencing mental health distress may have reduced emotional availability and are less likely to provide comprehensive care for a child. Since emotional availability and responsiveness are key components of secure attachments, the absence of these can worsen child EBDs. When caregivers struggle to provide stable emotional support, children may exhibit heightened anxiety, irritability, or behavioral dysregulation, which in turn increases the caregiving burden. This reciprocal cycle has been documented in prior research (Laurenzi et al., 2021) and is particularly salient in HIV-affected households where stressors such as stigma, economic hardship, and social isolation are common. While the cross-sectional design of this study limits causal inference, the findings align with theoretical and empirical evidence suggesting that child EBDs and caregiver mental health mutually reinforce one another over time within the family system. There is a need for culturally relevant and targeted interventions that aim at providing support to caregivers of children orphaned by HIV, which can be helpful to enhance positive mental health outcomes.

We found a significant association between caregiver gender and mental health outcomes particularly with male caregivers reporting poor mental health outcomes as compared to females. This finding emphasizes the influence of gender, caregiving and mental health outcomes as shown by existing literature (Bhan et al., 2020; Friedemann & Buckwalter, 2014; Neri et al., 2012). This difference may be understood within the sociocultural context of Uganda and similar SSA settings, where caregiving occurs alongside deeply entrenched patriarchal norms. One plausible explanation of the observed gender differences in mental health can be attributed to the highly patriarchal nature in the study region, which positions men as the breadwinners of the family (Schmidt-Sane et al., 2022). Men are often socially positioned as primary economic providers rather than nurturers, usually assuming intensive caregiving responsibilities, especially in the context of HIV-related settings, and may create role conflict and psychological strain (Okelo et al., 2022). Therefore, carrying the burden as the head of the family while also being a caregiver may increase the mental health challenges due to conflicts between caregiving duties and societal expectations. The dual role can exert pressure of financial provision and caregiving and may place male caregivers at heightened risk for mental distress While majority of the existing literature have demonstrated that women caregivers bear higher mental health difficulties as compared to men, the contrasting findings of this study may be explained by the traditional caregiving role which is often attributed to women (Okelo et al., 2022), leveraging on available resources and coping mechanisms making them exhibit better mental health outcomes compared to men. In contrast, women’s traditional socialization into caregiving roles may confer relative preparedness and access to informal support networks, as evidenced in the literature, suggesting that women are more likely to seek mental health support compared to men (Tedstone Doherty & Kartalova-O’Doherty, 2010). All these can buffer the mental health distress experienced by male and female caregivers. These findings indicate the need for gender-sensitive caregiving interventions that explicitly address men’s mental health, normalize help-seeking behaviors, and challenge restrictive gender norms that limit emotional expression and support utilization among male caregivers.

The significant positive association between family cohesion and caregiver mental health highlights the protective role of family cohesion in promoting positive mental health outcomes. among caregivers. Family cohesion is characterized by effective communication, supportive relationships, and mutual trust. A cohesive family may help caregivers manage the cumulative stress associated with caring for HIV-affected children with EBDs. This creates a positive, enabling environment that can help caregivers overcome life stressors and caregiving-related stress. In resource-limited settings, where formal mental health services are often scarce, family cohesion may function as a critical psychosocial resource. Our findings align with existing studies on family cohesion and caregivers’ mental health (Sousa et al., 2024; Trapp et al., 2019; White Makinde et al., 2024), indicating that family cohesion may buffer against stress and serve as a resource to alleviate the psychological burden associated with caregiving. Within HIV-impacted households, cohesive family structures may facilitate shared caregiving responsibilities, emotional validation, and collective coping, thereby reducing caregiver burnout. This finding shows the potential and the importance of family-level interventions that strengthen relationships and social functioning rather than focusing solely on individual caregivers or children. Another finding from this study is the significant association between alcohol use and poor caregiver mental health. Numerous studies have documented the impact of alcohol use on mental health (Hoare et al., 2021; Jardin et al., 2017; Milani & Perrino, 2021; Varma et al., 2024), echoing how alcohol use is linked to mental distress such as depression and anxiety. In the context of caregiving among children orphaned by HIV, alcohol use can either be a contributor or a consequence of mental health problems. Importantly, this association raises critical questions about the coping mechanisms caregivers employ when experiencing stress. As found by existing studies, caregivers may engage in alcohol use and other substance-using behaviors as a maladaptive behavior to cope with stress associated with caregiving (Rospenda et al., 2010). This can lead to further decline in their mental health as well as mental health problems in children. In addition, alcohol use can affect caregivers’ capacity to provide consistent care and capacity to provide consistent to children. Hence, the observed positive association between caregiver mental health and alcohol use may be explained as a caregiver coping mechanism to manage stress. Also, the effects of alcohol use on caregiver mental health and child difficulties should be considered in AIDS impacted families in SSA.

### Limitations

The study used self-reported data, which may be influenced by social desirability. Additionally, children’s difficulties were based on caregiver reports, which may be biased. The study did not consider important clinical variables for both children and caregivers, as well as school-related factors for the child, which could influence the association between child EBDs and caregiver mental health. Moreover, the findings are limited to caregivers who live with children who have been orphaned by HIV in rural Uganda, Masaka. Future research could explore these dynamics to identify the point of interventions in a wide ecological framework to improve child behavior problems, caregiver mental health and parenting among families affected by HIV. The results may not be generalized to the national population. This study used cross-sectional data; therefore, we recommend that future studies employ longitudinal designs to gain a more comprehensive understanding of the association between child behavior and caregiver mental health. This will enable researchers to track study subjects over a long period, providing insight into the long-term impact of interventions and understanding the patterns between these two factors. Finally, we acknowledge that school-related factors (including bullying, stigma, and academic difficulties) and clinical or familial factors could potentially influence the mental health of both children and caregivers. These represent important areas for future research, and we recommend that subsequent studies incorporate these variables to better elucidate their role in shaping mental health outcomes in this population.

## Conclusion

The study highlights the intrinsic relationship between child difficulties and caregiver psychological stress among families affected by HIV in Uganda. These findings indicate a necessity to develop comprehensive and multifaceted mental health interventions, such as psychoeducation, caregiver support groups, and stress management training, that address both child behavior and caregiver well-being rather than treating these issues individually or in isolation. The association between caregiver mental health problems and alcohol use also indicates the importance of incorporating substance-use screening and support into caregiver-focused programs. The observed association with alcohol use signals the need for interventions aimed at improving caregiver wellbeing to address substance misuse. Importantly, the protective role of family cohesion suggests that strengthening family relationships may buffer caregivers from experiencing mental health problems. In conclusion, the results emphasize the complex interplay of child difficulties, family cohesion, gender, and their influence on caregivers’ mental health, posing a significant implication for global health.

### Implications for Innovative Future Research and Interventions

The results indicate the complex interplay of child difficulties, family cohesion, gender, and their influence on caregivers’ mental health, posing a significant implication for global health. This indicates the need to build multi-component interventions or strategies to address caregiver mental health challenges in SSA, especially among those impacted by HIV. Building multi-component interventions to improve family cohesion and enhance caregiver mental health is much needed. For instance, interventions that improve families’ problem-solving and communication skills can strengthen family bonds, which in turn can limit children’s behavioral challenges.

Given the significant association between child difficulties and caregiver mental health, multi-component interventions that simultaneously support caregivers and strengthen family functioning are essential. These could include caregiver psychoeducation, family-based cognitive behavioral therapy (CBT), and structured family-strengthening programs that build communication, conflict resolution, and problem-solving skills. Such approaches have shown promise in low-resource settings and could be adapted for community delivery through lay counselors, village health teams, or school-based platforms. Enhancing family cohesion through these modalities may reduce caregiver stress and mitigate children’s behavioral challenges.

To advance research in this area, future studies should incorporate innovative and promising methodologies such as the Ecological Momentary Assessment (EMA) to capture real-time fluctuations in caregiver wellbeing (Moskowitz & Young, 2006; Shiffman et al., 2008). EMA is particularly well-suited to measuring variables such as daily stress levels, emotional reactivity, coping behaviors, and moment-to-moment caregiver–child interactions. These data would complement traditional cross-sectional and longitudinal designs by providing ecologically valid insights into how stress and emotional strain unfold in caregivers’ everyday environments. Such an approach could help identify high-risk periods or triggers for caregiver distress, informing more responsive and personalized intervention strategies.

Lastly, future research should also explore additional contextual factors, such as school-based stressors like bullying, that may contribute to children’s emotional and behavioral difficulties. Understanding these broader ecological influences will be critical for designing interventions that address both caregiver–child relationships and the wider social environments in which families operate.

## Figures and Tables

**Table 1:** Descriptive characteristics sample (*N* = 346)

Variable	*n* (%) / Mean (SD)
Age (min/max: 18–87)	45.78 (14.56)
Gender	
Male	71 (20.58)
Female	274 (79.42)
Household size (min/max: 1/12)	6.46 (2.28)
Total family assets (min/max: 0–15)	7.21 (2.95)
Social support network (min/max: 19–49)	37.55 (6.33)
Family cohesion	34.88 (4.10)
Cigarette use (yes)	23 (6.65)
Alcohol use (yes)	217 (62.72)
Child difficulties (min/max: 27–82)	51.19 (9.24)
Care giver’s mental health (min/max: 34–148)	73.60 (19.36)

**Table 2:** Bivariate analysis results: Associations between caregiver mental health and caregiver, household, and child characteristics

Variable	*r* / t-test	*p*-value
Age	*0.09*	*0.080*
Gender (t)	−*3.12*	*0.002*
Household size	−*0.06*	*0.292*
Total family assets	−*0.06*	*0.295*
Social support network	−*0.06*	*0.264*
Family cohesion	−*0.12*	*0.023*
Cigarette use (t)	−*0.01*	*0.990*
Alcohol use (t)	−*2.10*	*0.036*
Child difficulties	*0.37*	< *0.001*

**Table 3:** Mixed-effect regression model results for the association between the child’s emotional and behavioral difficulties and the caregiver’s mental health

Variable	β (95% CI)	*p* value
Age	<−0.001 (−0.14, 0.14)	0.997
Gender (ref: female)	**10.18 (5.65**, **14.72)**	**< 0.001**
Household size	−0.37 (−1.45, 0.70)	0.496
Total family assets	−0.47 (−1.25, 0.31)	0.234
Family cohesion	−**0.57 (−1.05**, −**0.09)**	**0.019**
Cigarette use (ref: yes)	3.39 (−9.23, 16.02)	0.599
Alcohol use (ref: yes)	**5.22 (2.03**, **8.41)**	**0.001**
Social support network	−0.01 (−0.33, 0.30)	0.939
Child difficulties	**0.85 (0.64**, **1.06)**	**< 0.001**
Constant	26.30 (−10.53, 63.13)	0.162
Variances		
School variance	0.453 (<−0.001 to 837980)	
Child variance	272.479 (224.679 to 330.448)	
ICC (school)	0.002 (< 0.001,1.00)	
ICC (participant)	0.994 (0.968, 1.00)	

* β unstandardized regression coefficient, *CI* confidence interval, *ICC* intraclass Correlation Coeefficient

## Data Availability

No datasets were generated or analysed during the current study.
